# Combined Proteomic and Metabolomic Analysis of the Molecular Mechanism Underlying the Response to Salt Stress during Seed Germination in Barley

**DOI:** 10.3390/ijms231810515

**Published:** 2022-09-10

**Authors:** Yiyou Chen, Juncheng Wang, Lirong Yao, Baochun Li, Xiaole Ma, Erjing Si, Ke Yang, Chengdao Li, Xunwu Shang, Yaxiong Meng, Huajun Wang

**Affiliations:** 1 Department of Crop Genetics and Breeding, College of Agronomy, Gansu Agricultural University, Lanzhou 730070, China; 2 State Key Lab of Aridland Crop Science/Gansu Key Lab of Crop Improvement and Germplasm Enhancement, Lanzhou 730070, China; 3 Department of Botany, College of Life Sciences and Technology, Gansu Agricultural University, Lanzhou 730070, China; 4 Western Barley Genetics Alliance, College of Science, Health, Engineering and Education, Murdoch University, Murdoch, WA 6150, Australia

**Keywords:** barley, abiotic stress, salt stress, proteomic, metabolomic

## Abstract

Salt stress is a major abiotic stress factor affecting crop production, and understanding of the response mechanisms of seed germination to salt stress can help to improve crop tolerance and yield. The differences in regulatory pathways during germination in different salt-tolerant barley seeds are not clear. Therefore, this study investigated the responses of different salt-tolerant barley seeds during germination to salt stress at the proteomic and metabolic levels. To do so, the proteomics and metabolomics of two barley seeds with different salt tolerances were comprehensively examined. Through comparative proteomic analysis, 778 differentially expressed proteins were identified, of which 335 were upregulated and 443 were downregulated. These proteins, were mainly involved in signal transduction, propanoate metabolism, phenylpropanoid biosynthesis, plant hormones and cell wall stress. In addition, a total of 187 salt-regulated metabolites were identified in this research, which were mainly related to ABC transporters, amino acid metabolism, carbohydrate metabolism and lipid metabolism; 72 were increased and 112 were decreased. Compared with salt-sensitive materials, salt-tolerant materials responded more positively to salt stress at the protein and metabolic levels. Taken together, these results suggest that salt-tolerant germplasm may enhance resilience by repairing intracellular structures, promoting lipid metabolism and increasing osmotic metabolites. These data not only provide new ideas for how seeds respond to salt stress but also provide new directions for studying the molecular mechanisms and the metabolic homeostasis of seeds in the early stages of germination under abiotic stresses.

## 1. Introduction

Salt stress is one of the foremost global environmental factors limiting plant growth and crop productivity [1,2]. Soil salinity is promoted by industrial pollution, poor irrigation practices and rising populations and is a major abiotic stress obstructing crop production especially in arid and semi-arid areas [2,3]. It is predicted that, by about 2050 and without the application of efficient management strategies, approximately 50% of arable soils will suffer from salinity [4]. One of the important salts in soil is sodium chloride, which has high solubility and ubiquitous distribution, and severe agricultural yield losses are caused by the effects of salinity factors on plant development and productivity [5,6]. Seed germination is considered the most critical stage in the life cycle and is highly susceptible to abiotic stresses, such as salt stress [7,8].

Seed germination refers to the process in which the metabolism is enhanced after seed imbibition, the key genes related to germination begin to be expressed, and the radicle gradually elongates and finally breaks through the endosperm and seed coat [9]. The inhibition of seed germination by salt stress is mainly manifested as osmotic stress, the accumulation of excessive reactive oxygen species, the destruction of the cell structure and the alteration of the phytohormone balance, which together reduce the germination rate and prolong the germination time [5]. Salt stress significantly affected seed germination by affecting various metabolic processes, including starch hydrolysis, sucrose transport and amino acid metabolism [10,11]. Thioredoxins (TRXs) are multifunctional proteins with catalytic activity that regulate sulfhydryl redox [12]. A thioredoxin protein-encoding gene *MsTRX* has been reported to improve salt tolerance by maintaining osmotic homeostasis in transgenic tobacco (*Nicotiana tabacum* L.) [13]. Abscisic acid insensitive 4 (ABI4), a key component of abscisic acid (ABA) signaling, is involved in reactive oxygen species (ROS) production and clearance and regulates ROS metabolism by directly binding to *RbohD* and *Vitamin C Defective 2* (*VTC2*) during the regulation of Arabidopsis (*Arabidopsis thaliana* L.) seed germination under salt stress [14]. A transcriptome study of Faba bean (*Vicia faba* L.) seed germination revealed that many differential genes involved in hormone metabolism (e.g., LEA gene), cell wall loosening and small interfering RNA pathways play an important regulatory role in seed germination under salt stress [15]. Glycinate betaine is an osmoprotectant of plants against abiotic stresses, and betaine aldehyde dehydrogenase (BADH), a key step in the biosynthesis of betaine glycinate, is overexpressed in plants to enhance their salt tolerance [16]. The plant response to salt stress is not determined by the proteins but also metabolites [17]. D-galactose [18], trehalose [19], raffinose [20,21], Myo-inositol [22,23], etc., are reported to be associated with salt tolerance in plants.

Tolerance to salt stress requires profound alterations to gene expression, which are accompanied by changes in the composition of the plant transcriptome, metabolome and proteome [24]. In recent years, the integration of various “omics” technologies has become an effective strategy to better understand the response mechanisms to environmental stress [25]. Data-independent acquisition (DIA), which involves sensitive protocols to perform and identify abiotic stress-responsive proteins in plants, has emerged as a powerful tool in quantitative proteomics [26,27]. Compared with previous proteomics methods DIA can provide a consistent, reproducible and accurate way to cover deeper data from multiple complex samples in shorter times [28,29]. Wide targeting of metabolite mechanisms by ultra-performance liquid chromatography–tandem mass spectrometry (UPLC-MS/MS) has been used to explore the ability of melatonin to alleviate the effects of drought on soybean growth [30]. Through the combined transcriptome and metabolome analysis of two millet genotypes with different tolerances under salt stress, the biosynthetic pathways of phenylpropanoids, flavonoids, lignin and lysophospholipids have been found to play important roles in determining the salt tolerance of millet [31]. The salt tolerance of tolerant varieties is mainly due to more efficient ion channels and antioxidant systems, which provide a comprehensive regulatory network for millet to handle salt, and the salt tolerance of other cereal crops provides some inspiration [31]. Based on the combination of omics approaches, more in-depth and systematic studies of the salt tolerance mechanisms of seed germination are possible.

Barley (*Hordeum vulgare* L.) is one of the most important cereals in the world, after wheat, maize and rice and is considered a marginal halophyte [32,33]. To investigate the differences in the regulation of salt response during germination of different salt-tolerant seeds, we selected two differentially salt-tolerant barley varieties, salt-tolerant variety GN2 and salt-sensitive variety GN18, which were identified in the previous laboratory screening. This study performed a comprehensive analysis of proteomics and metabolomics at 24 h of seed germination under salt stress to determine the changes in the regulation of their protein abundance and their metabolites. The results of this study provide new ideas for further research on the mechanism of salt tolerance during seed germination, help establish the salt response network of barley and provide candidate genes for the selection of new salt-tolerant varieties.

## 2. Results

### 2.1. Structural and Physiological Changes in Response to Salt Stress during Seed Germination

**Figure 1:**
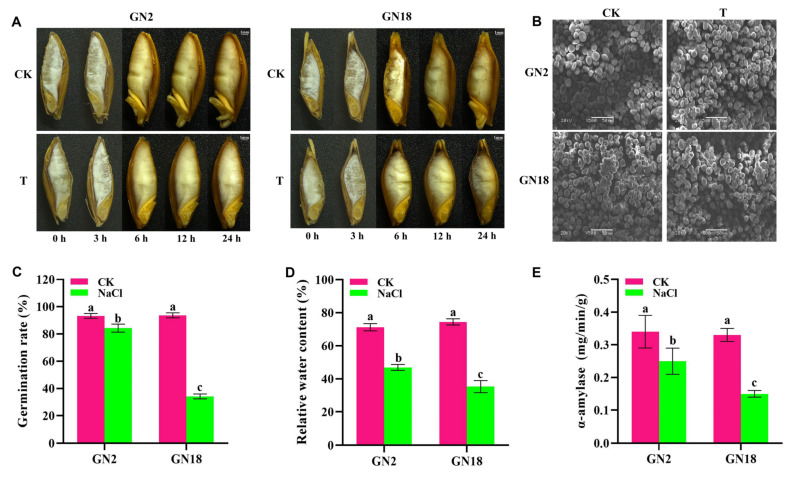
Morphological, ultrastructural and physiological parameter changes in the salt-tolerant barley cultivar GN2 and salt-sensitive barley cultivar GN18 during seed germination under salt stress. (**A**) Seed morphology images of seed germination from five periods in different treatment groups. (**B**) SEM images at 24 h of seed germination. (**C**) Seed germination rate. (**D**) Relative water content. (**E**) α-Amylase. Data are means three replicates (*n* = 3) ± standard deviation (SD). Different letters indicate significant difference at *p* < 0.05 as determined by one-way ANOVA test. CK, water-treated seeds; T, 200 mM salt solution-treated seeds; NaCl, 200 mM salt solution-treated seeds.

As shown in Figure 1, the radicle of seeds began to emerge at 6 h, and the size of the seeds increased gradually during the first 24 h of seed imbibition. The radicle growth was inhibited to various degrees after salt stress, especially in GN18 compared with GN2 (Figure 1A). The germination rate of GN18 was significantly reduced under salt stress (Figure 1C). Salt treatment had an effect the relative water content (RWC) during seed germination, and GN18 had lower RWC than GN2 between control and salt stress (Figure 1D). These results illustrated that GN2 was more tolerant to salt stress than GN18 during the seed germination stage. Ultrastructural observation under scanning electron microscopy showed that the two starch types, A and B, in seeds were oval and round, respectively. Under normal germination conditions, a concave texture appeared on the surface of the starch grains after seed imbibition, and starch degradation was activated. Under the of salt stress conditions, the size of the starch grains was clearly decreased, and the depression was not obvious. Compared with CK (water treatment), the numbers of small starch granules were increased in GN2 and GN18 after salt stress (Figure 1B). The activities of α-amylase had a decline to different degrees in both GN2 and GN18 after salt stress, but GN18 was more significantly affected (Figure 1E). This result indicated that starch degradation was activated during seed imbibition but that salt stress inhibited water uptake and starch degradation to a certain extent after 24 h of salt stress, resulting in delayed seed germination.

### 2.2. Analysis of DEPs in Seeds in Response to Salt Stress among Various Cultivars

**Figure 2:**
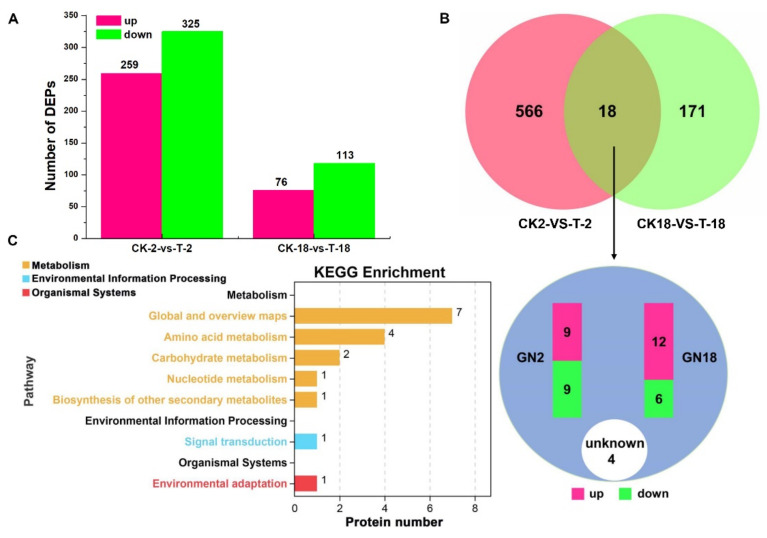
Differentially expressed proteins (DEPs) of different salt-tolerant cultivars in response to salt stress treatments during seed germination. (**A**) Number of DEPs in barley GN2 and GN18. (**B**) Venn diagram analyses of DEPs in seeds. The magnitude of the Rich Factor is positively correlated with the significance of the enrichment. (**C**) Visualization of common differentially abundant proteins between GN2 and GN18 and their KEGG pathway classification.

**Figure 3:**
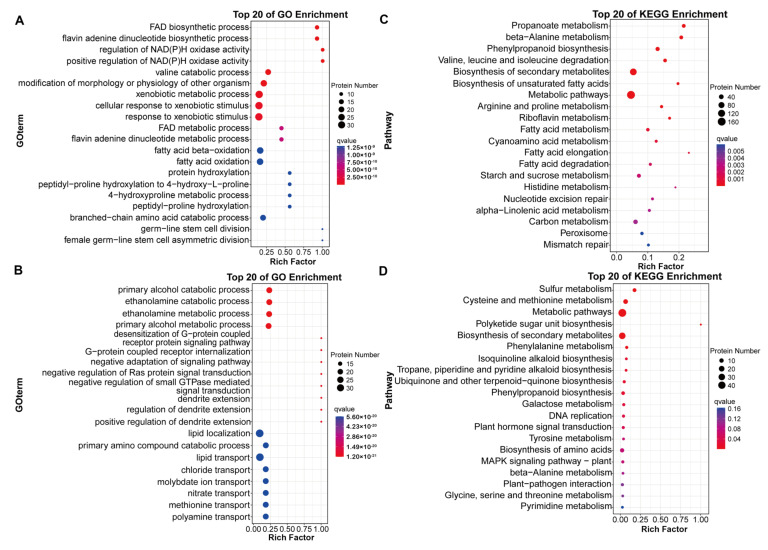
GO enrichment and KEGG pathway enrichment analysis of DEPs in GN2 and GN18 under salt stress at 24 h. Top 20 GO terms of the enriched scatter plot of DEPs in the salt response during the seed germination of (**A**) GN2 and (**B**) GN18. Top 20 KEGG pathways of the enriched scatter plot of DEPs in the salt response during the seed germination of (**C**) GN2 and (**D**) GN18.

To understand the effects of 24 h salt stress at the molecular level in seeds with different salt tolerances in this study, seeds germinated in distilled water and in 200 mM salt solution were investigated by data-independent acquisition (DIA) quantitative proteomics. In total, 18,640 proteins were identified and annotated in the seeds of GN2 and GN18 (Table S1). Among them, 778 proteins were regulated by salinity in GN2 and GN18 (Figure S1A,B, Table S2), of which 259 and 76 proteins were upregulated and 325 and 118 proteins were downregulated based the criteria: the ratios > 1.5 (upregulated) or  <  0.67 (downregulated) coupled with *p*  < 0.05, respectively (Figure 2A). Of these, there were 18 common differentially expressed proteins (DEPs) to GN2 and GN18 (Figure 2B). Gene ontology (GO) enrichment analysis was performed using the cluster profiler R package with a threshold value of *p* < 0.05, and the primary biological functions of the DEPs of GN2 and GN18 were classified into cellular components (CC, 450; 144), molecular function (MF, 458; 154) and biological process (BP, 490; 162) (Figure S1C,D). In GN2 and GN18, the biological process of the most DEPs were involved in metabolic process (18.68%, 17.51%, respectively), cellular process (17.83%, 14.87%, respectively), single-organism process (16.24%, 14.87%, respectively) and response to stimulus (9.36%, 8.51%, respectively) (Table S3). The most molecular functions were catalytic activity (41.61%) and binding (41.33%) in GN2, and the same molecular functions (42.96% and 35.56%, respectively) were also in GN18 (Table S3). For GN2, the GO terms mainly enriched were FAD biosynthetic process, flavin adenine dinucleotide biosynthetic process and regulation of NAD(P)H oxidase activity (Figure 3A). However, the GO terms mainly enriched were primary alcohol catabolic process, ethanolamine metabolic process, and negative adaptation of signaling pathway in GN18 (Figure 3B).

To further understand the characteristics of DEPs, analyses of the Kyoto Encyclopedia of Genes and Genomes (KEGG) pathway and protein domains were performed (Table S4). The results showed that the most significantly enriched pathways for GN2 included propanoate metabolism, beta-alanine metabolism, phenylpropanoid biosynthesis, fatty acid metabolism and valine, leucine and isoleucine degradation (Figure 3C). The DEPs were significantly enriched in 35 KEGG pathways in GN18 in cysteine and methionine metabolism, sulfur metabolism, phenylalanine metabolism and cysteine and methionine metabolism (Figure 3D). These results indicate that the different responses to salt stress between GN2 and GN18 at proteomic levels.

### 2.3. Metabolic Analyses of Barley Seeds in Response to Salt Stress during the Germination Process

**Figure 4:**
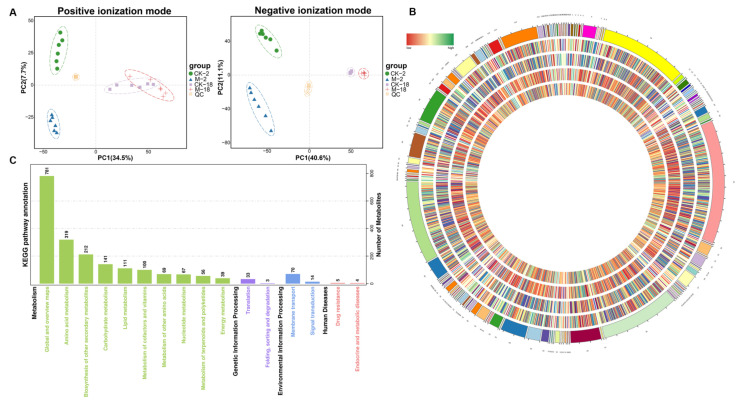
Metabolite profiles identified in GN2 (salt-tolerant) and GN18 (salt-sensitive) seeds with 24 h treatments involving water (CK) and 200 mM salt solution (salt treatment, M). (**A**) PCA of metabolomic profiles with quality control in positive and negative ionization modes. Overall score plot of seed samples collected at GN2 (CK-2 vs. M-2) and GN18 (CK-18 vs. M-18). (**B**) Circular diagram of all metabolites in the different groups, showing, from the outer circle to the inner circle, the metabolites of GN2 and GN18, respectively. (**C**) KEGG pathway enrichment analysis was performed for all identified metabolites.

**Figure 5:**
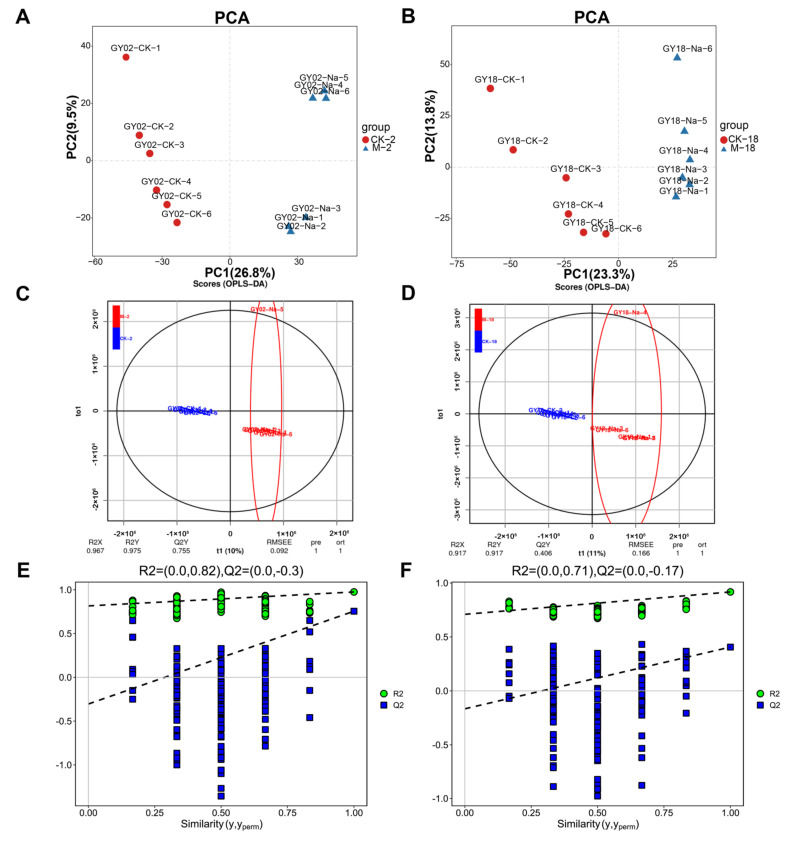
PCA score map (**A**,**B**), OPLS-DA scores (**C**,**D**) and permutation test (**E**,**F**) in positive ionization mode (POS). PCA score map of (**A**) GN2 and (**B**) GN18. (**C**) Scores of the OPLS-DA model in GN2 and (**D**) GN18. OPLS-DA cross-validation in (**E**) GN2 and (**F**) GN18. R^2^Y and Q^2^ denote the rate of the model interpretation of the Y matrix and the predictive ability of the model, respectively. Q^2^ > 0.9 indicates a good predictive model. The permutation test produces a distribution of R^2^^’^ and Q^2^^’^ values. A reliable model should produce much larger values of R^2^^’^and Q^2^^’^ than a random model using the same dataset. Green and blue points indicate R^2’^ and Q^2^^’^ values, respectively.

To interpret the major effect of 24 h salt stress on GN2 and GN18 seeds germination at the metabolite level, we performed principal component analysis (PCA) on seeds and quality control (QC) samples of GN2 and GN18 to monitor the accuracy and repeatability of the analytical process. Regarding positive ionization (PI) mode datasets, 34.5% and 7.7% were explained by the first principal component (PC1) and the second principal component (PC2), respectively (Figure 4A). Similarly, PC1 and PC2 accounted for 40.6% and 11.1% of the total variability of the negative ionization (NI) model dataset, respectively. A total of 12,402 metabolites were identified during the analysis (Table S5). All known metabolites included 1013 of positive ionization mode (POS) type and 1014 of negative ionization mode (NEG) type, which were classified into 137 categories, with the dominant categories being prenol lipids, steroids and steroid derivatives, fatty acyls, organooxygen compounds, benzene and substituted derivatives and carboxylic acids and derivatives (Figure 4B, Table S6). Metabolites production was mainly enriched in global and overview maps (781), amino acid metabolism (319) and biosynthesis of other secondary metabolites (212) (Figure 4C). The metabolites of GN2 and GN18 samples were well separated along PC1 revealed by PCA (Figure 5 and Figure S2). Orthogonal projection to latent structures-discriminant analysis (OPLS-DA) is the model that maximizes the benefit of viewing differences between groups. The R^2^ values all exceed 0.9 for GN2 and GN18 (Figure 5). In addition, the OPLS-DA model was validated by permutation tests using 100 alignment experiments. The replacement R2’ and Q2’ values were less than the corresponding R^2^ and Q^2^ values of the original model. Therefore, differential accumulated metabolites (DAMs) could be screened between control and treatment by the model with variable importance for projection (VIP) ≥ 1 and that the *t*-test *p* < 0.05.

**Figure 6:**
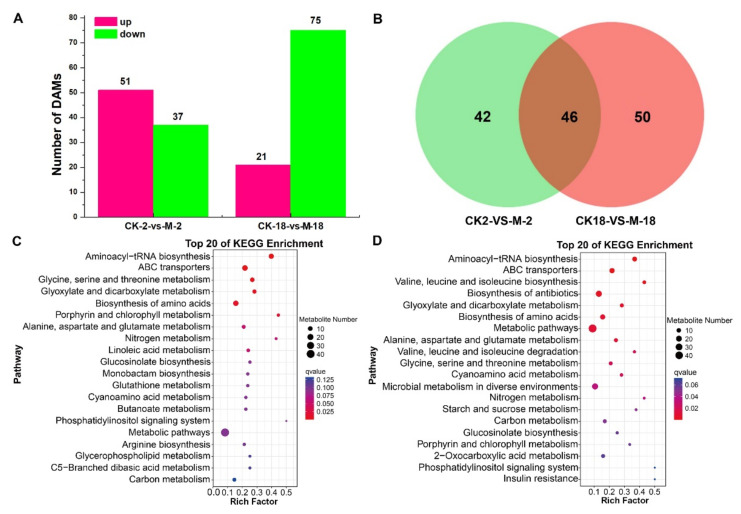
Differentially accumulated metabolites (DAMs) in GN2 and GN18 in response to salt stress treatments. (**A**) Numbers of differentially accumulated metabolites in barley GN2 and GN18 for 24 h. (**B**) Venn diagram analyses of DAMs in seeds. KEGG pathway enrichment analysis of DAMs in (**C**) GN2 and (**D**) GN18.

In total, we identified 184 differential accumulated metabolites in GN2 and GN18, of which 25.0%, 16.8% and 8.7% were carboxylic acids and derivatives, organooxygen compounds and fatty acyls, respectively (Table S7). Compared with the control seeds of GN2 (CK-2) and GN18 (CK-18), the number of DAMs for the 200 mM salt solution-treated seeds of GN2 (M-2) and GN18 (M-18), respectively, comprised 88 (51 upregulated; 37 downregulated) and 96 (21 upregulated; 75 downregulated) (Figure 6A, Table S7). Venn diagram analysis showed 46 common metabolites between GN2 and GN18, as well as 42 and 50 specific differential accumulated metabolites, respectively (Figure 6B, Table S8). KEGG pathway enrichment analysis was performed to reveal the most important pathways related to the responses of the different varieties to salt stress (*p* < 0.05) (Table S9). Compared with the salt-treatment group, the DAMs of GN2 were mainly involved in aminoacyl-tRNA biosynthesis, ABC transporters, glycine, serine and threonine metabolism, glyoxylate and dicarboxylate metabolism and porphyrin and chlorophyll metabolism (Figure 6C), whereas the DAMs of GN18 were mainly involved in valine, leucine and isoleucine biosynthesis, biosynthesis of amino acids, alanine, aspartate and glutamate metabolism, glycine, serine and threonine metabolism and cyanoamino acid metabolism (Figure 6D). These results investigated that the changes of these accumulated metabolites and metabolic pathways provide essential information on differences in salt tolerance among genotypes.

### 2.4. Integrative Proteomic and Metabolomic Analyses

**Figure 7:**
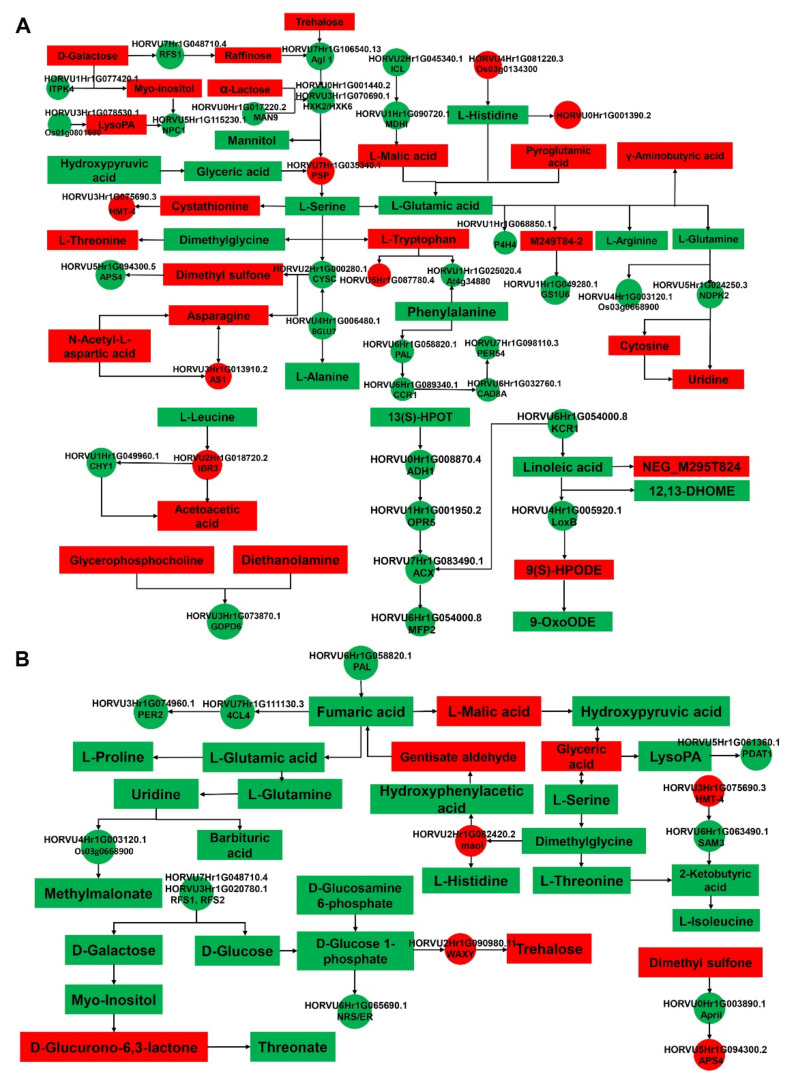
Biological response pathways of GN2 (**A**) and GN18 (**B**) seeds in response to salt stress. Correlation analysis results of DEPs and DAMs were mapped into a comprehensive metabolic regulation network diagram based on the KEGG pathway. The DEPs and DAMs are marked as circles and rectangles, respectively; red and green indicate upregulation and downregulation, respectively.

**Figure 8:**
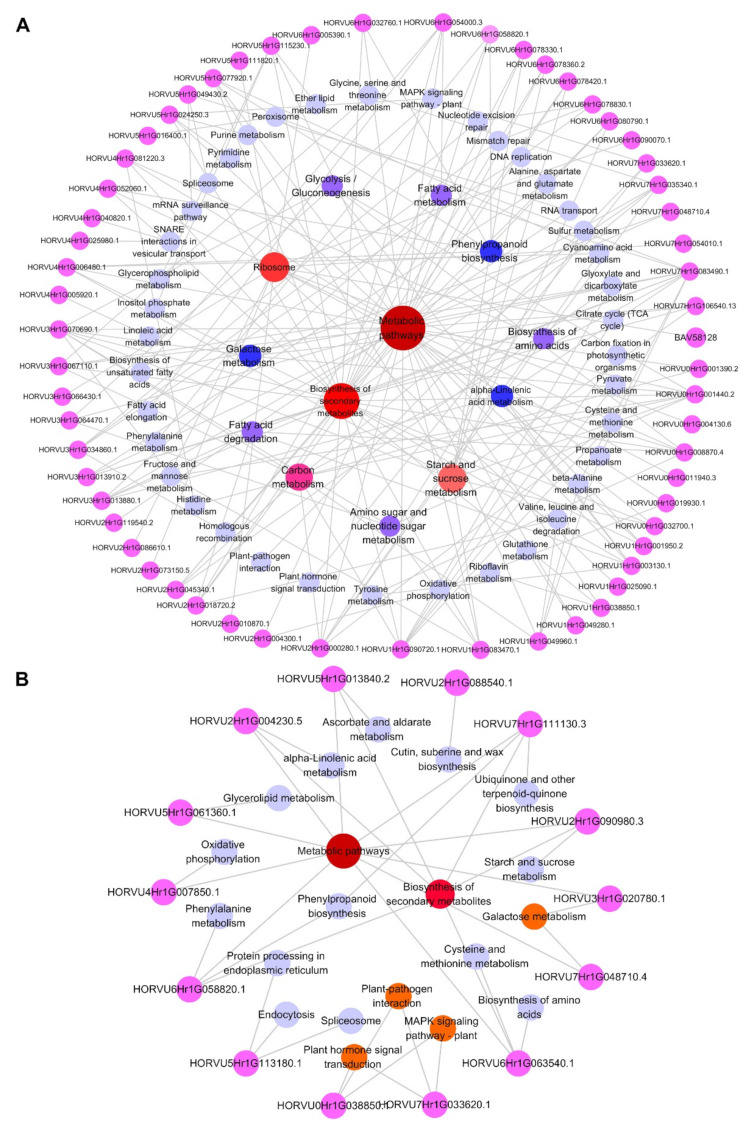
A summary of the partially critical DEPs related to the response of GN2 (**A**) and GN18 (**B**) seeds to salt stress and the KEGG pathways involved. All proteins are represented by a pink circle, and the KEGG pathways are represented by circles of different colors. The color shade and circle size are positively correlated with the number of proteins contained in the circle, with the least amount of protein contained in purple circles and the most amount of protein contained in red circles.

Correlation analysis between DEPs and metabolites under salt stress revealed that 47 and 22 metabolic pathways were enriched in GN2 and GN18, respectively (Table S10). The metabolic pathways of GN2 included phenylpropanoid biosynthesis, propanoate metabolism, cyanoamino acid metabolism, starch and sucrose metabolism and biosynthesis of unsaturated fatty acids. Meanwhile, sulfur metabolism, cysteine and methionine metabolism, phenylalanine metabolism, tropane, piperidine and pyridine alkaloid biosynthesis and galactose metabolism were identified in GN18. However, the core DEPs and DAMs regulatory networks of the two different genotypes included 36 and 22 metabolic pathways, respectively, indicating the response of different salt-tolerant barley seeds to salt stress (Table S10). To comprehensively assess potential molecular mechanisms of salt stress response in two genotypes, we mapped the comprehensive systemic biological pathway through the analysis KEGG pathways of DEPs and DAMs (Figure 7). There were 36 proteins and 39 metabolites in GN2 (Figure 7A), 13 proteins and 28 metabolites in GN18 (Figure 7B). We noticed that the accumulation of L-histidine, L-serine and L-glutamic acid, hydroxypyruvic acid, trehalose and L-malic acid were the same in both genotypes. However, the accumulation of L-threonine, D-galactose, myo-Inositol, LysoPA and uridine were upregulated in GN2, whereas they were downregulated in GN18. Throughout the biological process, more DEPs were involved in the regulation of GN2 compared with GN18 may have contributed to these results (Figure 8). These results suggested that the expression of specific proteins and the accumulation of metabolites under the same metabolic pathway, and the regulation of specific metabolic pathways lead to the differences in salt tolerance between the two cultivars.

## 3. Discussion

As a major adverse environmental factor, salt stress greatly affects crop yields worldwide [34]. Crops have evolved elaborate regulatory mechanisms to manage salt stress, including modifications to protein composition and regulatory activities at the overall level as well as in changes in metabolites [35,36]. In this study, the alteration of proteins and metabolites of two types of barley seeds with different salt tolerances was studied after germination for 24 h under salt stress.

### 3.1. Signal Transduction in Response to Salt Stress

Signal transduction pathways play an important role in plant responses to abiotic stresses [37]. Calcium, which is key to the regulation of plant growth and development by Ca^2+^-modulated proteins, is involved in a variety of cellular processes [38]. During salt stress, Ca^2+^ signaling, whose function and consequence are not limited to the single-cell level, representing a complex phenomenon [39]. Wan et al. found that calcium-transporting ATPase in the Ca^2+^ signaling pathway responds to salt stress by upregulating its expression [40]. In our study, calcium-transporting ATPase (HORVU6Hr1G030590.12) was upregulated in GN2 after salt stress during seed germination at 24 h, demonstrating that this protein plays a positive regulatory role in the salt stress response. ROPs, small monomeric GTPases, are signaling hubs that regulate some cell polarity processes, usually those involving cytoskeleton reorganization, and are identified as molecular switches to shuttle between a signaling-inactive or activated GTP-bound state [41,42]. Guanine nucleotide exchange factor-mediated of ROPs in turn regulates plant responses to complex environmental conditions through reproduction [43]. Rop guanine nucleotide exchange factor 1 (RopGEF1) (HORVU3Hr1G085680.4) is upregulated in salt-tolerant material, which might be related to the salt stress response (Table S2). In addition, RopGEF1 can be considered a negative regulator of the phytohormone ABA in signal transduction [44]. Taken together with our findings, these results will provide the basis for further research into the function of RopGEF1 in salt tolerance. Many ubiquitin-like proteins (UBLs) have been found in almost all eukaryotic organisms [45]. Although they are involved in a large number of physiological processes [46], there are few reports on the function of UBL5 in plants, particularly with regard to unsatisfactory environmental conditions. However, more and more studies have examined the UBL5-mediated plant response to biotic and abiotic stress in recent years. UBL5 might participate in the regulation of plant defense against pathogens [46]. UBL5 overexpression in transgenic perennial ryegrass (*Lolium perenne* L.) plants improves drought tolerance, which improves drought tolerance characterized by higher leaf water potential and relative water content [47]. UBL5 (HORVU5Hr1G042600.1) was found to be upregulated after salinity stress in the salt-tolerant cultivar in this study (Figure 7, Table S2). This result suggests that UBL5 may have a salt-tolerant function in plants.

### 3.2. Amino Acid Metabolism for Salt Tolerance

**Figure 9:**
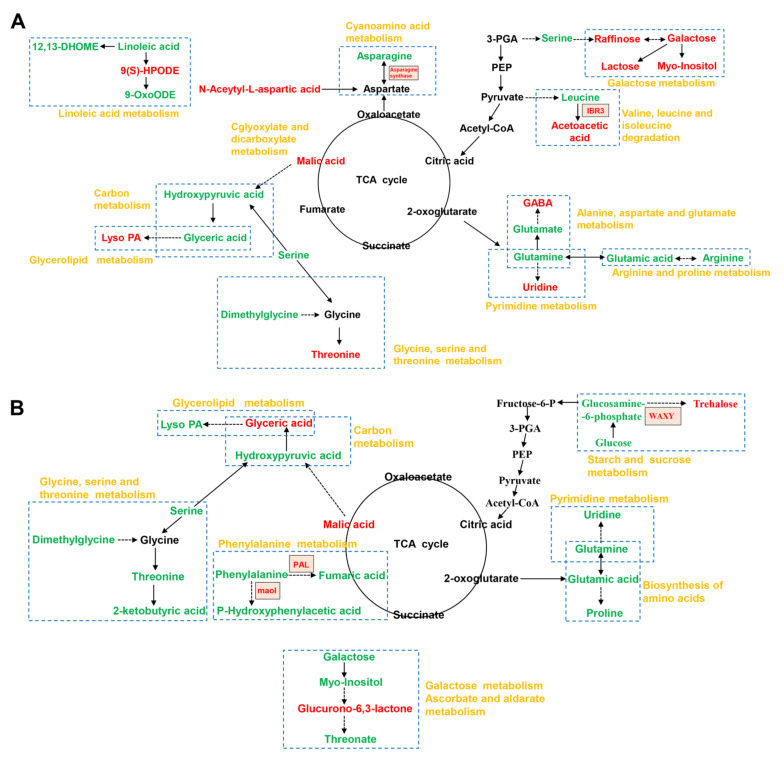
The network of key DAMs in GN2 (**A**) and GN18 (**B**) seeds induced under salt stress in metabolism. The upregulated and downregulated metabolites are indicated in red and green colors, respectively. The metabolic pathway is circled with a blue dotted border, and the name of the metabolic pathway is written in yellow font. The dotted arrows indicate multiple steps between the two metabolites.

Amino acid metabolism is one of the critical ways through which plants can respond to salt stress [48]. Glycine, serine and threonine metabolism play a crucial role in salt tolerance [48]. Betaine aldehyde dehydrogenase is an important gene involved in glycine betaine biosynthesis pathway, and its introduction can enhance the tolerance of plant to various abiotic stresses [49]. In this study, the regulation of DEPs and DAMs in this pathway was different in the two genotypes. We detected that L-threonine was increased in GN2 but decreased in GN18, and glyceric acid upregulated in GN18 but downregulated in GN2 (Figure 9). A unique metabolite 2-Ketobutyric acid was detected in GN18 (Figure 9). In addition, betaine aldehyde dehydrogenase (HORVU2Hr1G080970.14), glycine cleavage system P protein and a predicted protein were regulated in GN2 among this pathway, but only amine oxidase (HORVU2Hr1G082420.2) was regulated in GN18 (Table S4). In addition, the contents of metabolites in GN2 related to the salt stress response, such as threonine, asparagine, gamma-aminobutyric acid, gamma-glutamylcysteine and trans-Aconitate, were significantly increased, while the accumulation of phenylalanine, glutamic acid, arginine, glutamine, beta-tyrosine and histidine was decreased to varying degrees (Figure 7A, Table S7). In contrast, creatinine, gamma-aminobutyric acid and gamma-glutamylcysteine accumulated significantly in GN18, whereas the accumulation of phenylalanine, histidine, threonine, glutamic acid and proline was significantly decreased (Figure 7B, Table S7). Interestingly, we made the same result prediction as Pan et al. [31]. We speculated that the high tolerance of GN2 may be related to its ability to maintain intracellular homeostasis through the accumulation of amino acids and the further use of synthetic amino acids for the formation of the carbon skeleton of the cell membrane and the synthesis of secondary metabolites.

### 3.3. Carbohydrate Metabolism for Salt Tolerance

Higher accumulation of carbohydrates, the main source of energy stored in plant vegetative organs, in plants after stress indicates better tolerance [50,51]. Carbohydrates include sugars and sugar alcohols such as raffinose, trehalose, galactose, glucose, and fructose [52]. D-galactose is sugar moiety linked to mature N-glycoprotein, and mature N-glycoprotein may affect the ability of plants to adapt to salt stress, which has been confirmed in Arabidopsis and Solanum lycopersicum [18,53,54]. Raffinose belongs to the raffinose family of oligosaccharides (RFOs) and is an osmoprotectant that can accumulate under abiotic stress and is the key to plant defense mechanisms [20,21]. We found that D-galactose, raffinose and alpha-lactose rapidly accumulated in GN2 after being induced by salt stress (Figure 7A and Figure 9A, Table S7). However, in GN18, the accumulations of D-galactose, D-glucose and D-glucose 1-phosphate were all decreased (Table S7). In the galactose metabolism pathway, although probable galactinol--sucrose galactosyltransferase 1 (HORVU7Hr1G048710.4) was downregulated in both cultivars, the downregulation of seed imbibition protein (HORVU3Hr1G020780.1) in GN18 may be the responsible reason for the difference in salt tolerance between species (Table S4). Polyols can prevent metabolic inactivation under low osmotic conditions due to their water-like hydroxyl groups and can act as osmotic protective agents [55]. Myo-inositol is the most reported polyol, and an increase in myo-inositol metabolism attenuates salt-induced damage [22,23]. After 24 h of salt stress in seeds, the myo-inositol content was significantly increased in GN2, whereas the myo-inositol synthesis of GN18 was significantly decreased. Therefore, we speculate that GN2 can increase salt tolerance by rapidly inducing the metabolism of soluble sugar, accelerating the biosynthesis of myo-inositol, and improving the osmotic balance and antioxidant system activity under salt stress.

### 3.4. Effects of Salt Stress on Phytohormone Signal Transduction

Endogenous plant hormones are closely related to plant growth and development and play an important role in oxidative stress. ABA fulfils a pivotal regulatory role in the plant abiotic stress response and is the main hormone regulating seed germination, which can alleviate the impact of salt stress on seed germination [56,57]. Salt stress triggers a rapid accumulation of ABA in plants in a short period of time and induces some ABA-induced proteins [58,59]. Late embryogenesis-abundant (LEA) proteins are involved in the ABA signal transduction process [60]. OsLEA5 is validated to be involved in ABA-mediated antioxidant defense and plays a role in rice drought and salt stress responses [61]. Previous studies have shown that LEA protein belongs to a small family of highly hydrophilic proteins that respond to salt stress environments and that are induced by osmotic stresses in vegetative tissues, boosting plant tolerance to osmotic and oxidative stress [62,63]. We found that LEA proteins (HORVU1Hr1G059910.1) were significantly upregulated in GN2 and involved in numerous biological processes, including the ABA response, postembryonic development and seed development. It is worth mentioning that LEA protein (HORVU1Hr1G059900.1, HOR-VU3Hr1G030650.1) was also identified in GN18, but the upregulated expression fold change was less than that of GN2 (Table S2). Early methionine (Em) protein, characterized as group 1 LEA protein, was first identified and described in wheat embryos and is reported to play a fundamental role in the response to environmental stress [64,65]. Em protein CS41, which is significantly expressed in barley seeds after 24 h and 48 h of salt stress, can respond to ABA [66]. Our results further confirm this conclusion. The expression of Em protein CS41 (HORVU1Hr1G059950.2) was significantly upregulated after 24 h stress in salt-tolerant materials. Similarly, dehydration proteins (dehydrins), which can be significantly upregulated by abiotic stresses such as salinity, have been identified as a group 2 member of the LEA family, which accumulate abundantly after salt stress during seed germination [67,68]. The 20 dehydrins, in GN2 were significantly upregulated after 24 h of salt stress during seed germination, which may enhance salt tolerance by retaining water in the seeds and thus alleviating salt stress. One negative regulator of the ABA signaling pathway is the nodulin homeobox (NDX) protein, which is inhibited by ABA to enhance ABI4-mediated inhibition of storage reserve mobilization and germination [69,70]. In our research, the NDX-like proteins were significantly upregulated in salt-tolerant materials, presumably because NDX inhibited ABI4 expression and thereby alleviated ABI4-mediated mobilization of storage reserves and inhibition of seed germination (Table S2). The specific expression of NDX may also be an important regulatory mode in the mechanism of GN2 salt tolerance. An enormous range of BTB protein family members, such as BTB domain-containing protein, is confirmed to participate in biological regulatory networks and regulate hormone-mediated signaling in plants under abiotic stress [71,72]. The RCAR-PP2C-SnRK2 regulatory module can mediate reversible protein phosphorylation regulation, enabling ABA to play a regulatory role in plant abiotic stress responses [73,74]. In this process, PP2C inhibits the activity of SnRKs through negative regulation, leading to a signaling cascade reaction [56,75]. PP2C (HORVU2Hr1G046600.4) was upregulated in GN18 but was not identified in GN2. Thus, we speculate that ABA signaling may have more complex and diverse regulatory patterns in the first 24 h of seed germination.

### 3.5. Cell Walls, Biomembranes and Protein Stability under Salt Stress

The cell wall plays a critical role in protecting plants from abiotic stresses [76]. Leuine-rich repeat extension (LRX) protein acts as a cell wall localization protein important for regulating cell wall integrity [77]. There are 11 LRX proteins in total, which can generally be divided into two clades according to their tissue-specific expression patterns, with LRX1–LRX7 mainly expressed in vegetative tissues [78,79]. It would be an interesting re-search direction to elucidate how LRXs participate in the sensing of cell wall integrity [76]. The upregulated LRX proteins (HORVU6Hr1G089280.1) in GN2 may improve the salt tolerance of seeds (Table S2). We will further study these proteins in the future. The genes encoding transmembrane proteins are associated with salt tolerance [80]. Transmembrane protein (HORVU4Hr1G050360.1) was identified as upregulated in GN2. Non-specific phospholipase (NPC), also known as phosphatidylcholine-specific phospholipase C (PC-PLC), plays an important role in response to abiotic stresses [81]. We found NPC1 (HORVU5Hr1G115230.1) was regulated in GN2 seeds under salt stress (Figure 7, Table S2). Furthermore, we identified lysophosphatidic acid (lysoPA), which was relative to salt tolerance both in GN2 and GN18, within different accumulation ways (Figure 7) [82]. The changes in the fatty acid composition of membrane lipids are closely related to the salt tolerance of plants [83]. A reduction in saturated fatty acid contents and a high level of unsaturated fatty acids can preserve the membrane fluidity necessary for membrane function, which is an effective way for plants to adapt to salt stress [84]. Linoleic acid, an unsaturated fatty acid, is involved in basal energy metabolism and lipid signal transduction in the abiotic stress response, and plays a variety of roles in plant development and the stress response [85,86]. In our study, linoleic acid synthesis was inhibited in both materials with different salt tolerances but particularly in salt-sensitive materials. In addition, fatty acyls, including 9(S)-HPODE, 13S-hydroxy octadecadienoic acid and 11-dehydrothromboxane B2, were rapidly accumulated after salt induction in GN2 (Figure 7A, Table S7), whereas only erucic acid accumulated in GN18 (Figure 7B, Table S7). We noted a significant difference in the accumulation of 13S-hydroxylinoleic acid in the two materials: while it was rapidly induced in GN2, its synthesis was significantly inhibited in GN18. We speculate that one of the reasons for the higher salt tolerance of GN2 compared to GN18 is its ability to maintain a considerable level of unsaturated fatty acids that can reduce the effect of salt damage on the membrane. Ribosomal protein (RP) is an essential component of the ribosome that is responsible for protein synthesis [87,88]. RPs are transcriptionally affected under abiotic stress conditions, which significantly influences the translation of other proteins and to regulate protein synthesis in response to osmotic stress [89,90]. A total of 24 RP-like DEPs were detected in our study, of which 14 were upregulated and 20 were downregulated. Among salt-tolerant materials, we detected 14 upregulated and 8 downregulated DEPs, and 2 were downregulated in salt-sensitive materials (Table S2). Previous studies found that RPS4, RPS6, RPS26, RPS29 and RPL37 were upregulated in response to stress to enhance plant stress resistance, which is consistent with our findings [91,92].

### 3.6. Antioxidants under Salt Stress

Saline stress in plants will lead to a breakdown of the balance between ROS production and scavenging, resulting in changes in intracellular homeostasis, increased plasma membrane oxidation and increased saturated fatty acids [93,94]. To detoxify, plants have evolved ROS scavenging systems that involve enzymes [95]. Thioredoxins (TRXs) are involved in the regulation of the cellular redox environment and play an important role in complex redox regulation in response to environmental signals, protein transcription and translation, seed germination, cell division and development [96,97]. Chloroplastic thioredoxins are involved in the light regulation of carbon metabolism by regulating the pentose phosphate pathway and the C4 pathway [96]. TRXs in plants are classified into different types, and y-type TRXs are chloroplastic [98,99]. We found that five y-type TRX proteins were upregulated in GN2 (Table S2). Hence, we speculate that y-type TRXs, as the main antioxidants in plastids, may have physiological significance and new functions in salt resistance in barley seeds. Protein L-isoaspartate O-methyltransferase (PCMT1 or PIMT) is a widely distributed and highly conserved enzyme related to protein repair functions [100,101,102]. The researchers found that overexpression of OsPIMT1 and OsPIMT2 in rice seeds significantly reduced the contents of hydrogen peroxide and malondialdehyde in the seeds and correspondingly increased the contents of catalase and ascorbic acid peroxidase [103]. In the present study, PCMT (HORVU2Hr1G083100.1) was found to be upregulated in GN18 (Table S2). To the best of our knowledge, this is the first time that these proteins have been identified in barley seeds.

## 4. Materials and Methods

### 4.1. Barley Materials and Seed Germination under Salt Stress

GN2 (salt-tolerant) and GN18 (salt-sensitive) barley land cultivars were obtained from State Key Lab Key Lab of Aridland Crop Science/Gansu Key Lab of Crop Improvement and Germplasm Enhancement. The germination experiment consisted of two treatments, distilled water as the control and salt stress treatment using 200 mM NaCl solution. Each 250 grains of both barley cultivars were first washed with 75% alcohol for 15 s and then washed three times with sterile deionized water and were placed in 15 cm Petri dishes that contained two pieces of filter paper in the dark at 22 °C for 24 h. The seeds were subsequently stores at −80 °C until their analysis.

### 4.2. Measurement of Seed Morphology and Physiological Parameters

Seed morphology was investigated and photographed with a stereomicroscope (Leica-M165 C; Leica, Wetzlar, Germany). With reference to Sangwongchai et al. [104], different grain ultrastructures from various cultivars were visualized using scanning electron microscopy (SEM). Each treatment had three biological replications. Seed germination was defined as radicle protrusion. The seed germination rate was calculated by counting the number of germination of 50 seeds in 124 h with three biological replicates for each treatment. In this experiment, the method of determining the relative water content (RWC) of Mostofa [105] was adapted and improved. The relative water content (RWC) was calculated using the weight of freshly collected seeds and the weight of seeds after drying to a constant weight in an oven at 105 °C. The formula was: RWC = (FW − DW)/DW × 100. The α-amylase activity was measured by using the assay kits (Laierbio, Hefei, China, LE-Y1795) according to the manufacturer’s protocols.

### 4.3. Isolation of Total Proteins and Proteome Analysis

Total proteins from each sample (CK-2, GN2 treated with water; T-2, GN2 treated with 200 mM NaCl; CK-18, GN18 treated with water; T-18, GN18 treated with 200 mM NaCl) were extracted using the cold acetone method [106]. The total extracted protein concentration was determined using a BCA protein assay kit. Proteins were then digested with sequence-grade modified trypsin (Promega, Madison, WI, USA). The peptide mixture was redissolved in buffer A (20 mM ammonium formate in water, pH = 10.0, adjusted with ammonium hydroxide), and each peptide mixture sample was fractionated by high pH separation using an Ultimate 3000 system (ThermoFisher scientific, Waltham, MA, USA) connected to a reverse-phase column (XBridge C18 column, 4.6 mm × 250 mm, 5 μm, (Waters Corporation, Milford, MA, USA). For subsequent identification, 10 separated fractions were collected and dried for each sample. In addition, collected peptide fractions were analyzed by online nanospray LC-MS/MS on an Orbitrap Fusion Lumos coupled to an EASY-nLC 1200 system (Thermo Fisher Scientific, Waltham, MA, USA). Analyses of data-dependent acquisition (DDA) and data-independent acquisition (DIA) proteomics were performed with three biological replicates. Raw DDA and DIA data were processed and analyzed using Spectronaut X (Biognosys AG, Schlieren, Switzerland) with default settings and parameters. The Spectronaut was set up to search the database of barley along with the contaminant database, assuming the use of trypsin as the digestion enzyme. The retention time prediction type was set to dynamic iRT. Data extraction was determined by Spectronaut X based on extensive mass calibration. Spectronaut Pulsar [107] will dynamically determine the ideal extraction window dynamically depending on the iRT calibration and gradient stability. A Qvalue (FDR) cutoff of 1% was applied to the precursor and protein level. Decoy generation was set to mutated, which is similar to scrambled but will only apply a random number of amino acid position swamps (min = 2, max = length/2). All selected precursors passing the filters were used in the quantification. Proteins were annotated by the Gene Ontology (GO) and Kyoto Encyclopedia of Genes and Genomes (KEGG) databases, with a fold change >1.5 or <0.67 and Q value < 0.05 considered to indicate differentially expressed proteins (DEPs) within the functional enrichment of the GO term annotation and KEGG pathway. Protein–protein interaction (PPI) network was identified using String [108] and the network file was visualized using Cytoscape software [109].

### 4.4. Detection and Identification of Metabolites and Analysis

The total metabolites in the samples were extracted according to previous studies [110,111]. Briefly, 100 mg of lyophilized sample was prepared by using a grinder at 30 Hz for 1.5 min. The sample was then homogenized with 1 mL of pre-chilled methanol (−20 °C) for 1 min. Afterward, the samples were centrifuged at 13,000× *g* for 15 min at 4 °C and the obtained supernatants were absorbed and filtered prior to UPLC-MS/MS analysis [112]. The quality control (QC) sample was prepared by a mixture of equal amounts of the supernatants from all of the samples. A UHPLC system (Thermo UltiMate 3000) with a UPLC HSS T3 column (2.1 mm × 150 mm, 1.8 μm) coupled to a Q Exactive system (Orbitrap MS, Thermo) was used for LC-MS/MS analyses according to the method described by Wang et al. [113]. The acquired raw MS data files were converted to mzXML format using Proteowizard (v3.0.8789). Then, data peak identification, filtration and alignment were performed with the R statistical package XCMS (v3.1.3). [114]. For a visualization of the differences among different groups of samples, the identified metabolites were subjected to principal component analysis (PCA), an unsupervised dimensionality reduction method, to partial least squares discriminant analysis (PLS-DA), a supervised dimensionality reduction method and to orthogonal projection to latent structures-discriminant analysis (OPLS-DA) using the corresponding R package models (http://www.r-project.org/, accessed on 20 August 2015). Metabolites compared between two groups with variable importance of the projection (VIP) ≥ 1 and *p* < 0.05 based on *t*-test were used to identify differentially accumulated metabolites (DAMs). Furthermore, DAMs were mapped to the online KEGG software for pathway enrichment analysis (FDR ≤ 0.05).

## 5. Conclusions

In the present study, we used DIA and untargeted LC/MS to reveal the molecular mechanisms of different salt-tolerant barley during seed germination in response to salt stress. In total, 778 DEPs and 187 DAMs were identified after 24 h salt stress. Further analysis of proteomic and metabolomic investigations indicated that the differences in salt tolerance among various cultivars were related to the relevant metabolic pathways involved in the TCA cycle. These results provide a basis for further elucidation of the mechanisms of barley seed germination in response to salt stress and provide an important theoretical starting point for resolving the mechanisms of seed germination in response to abiotic stresses.

## Data Availability

The mass spectrometry proteomics data have been deposited in the ProteomeXchange Consortium (http://proteomecentral.proteomexchange.org) via the iProX partner repository [115] with the dataset identifier IPX0004221003/PXD032701.

## Supplementary Materials

The supporting information can be downloaded at: https://www.mdpi.com/article/10.3390/ijms231810515/s1.
