# Supplementary figures and images for: Combined Proteomic and Metabolomic Analysis of the Molecular Mechanism Underlying the Response to Salt Stress during Seed Germination in Barley
Source: Int J Mol Sci. 2022 Sep 10;23(18):10515. doi: 10.3390/ijms231810515 (PMC9499682; doi:10.3390/ijms231810515)

**A**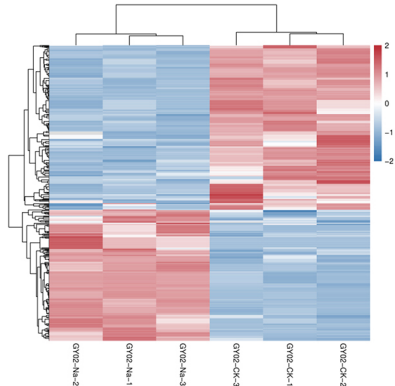**B**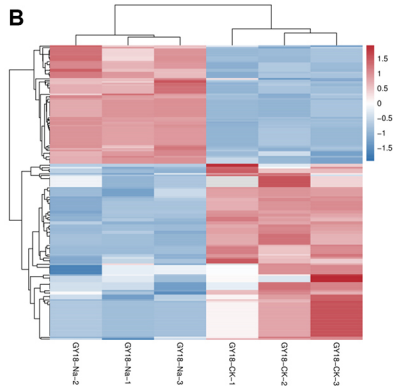**C**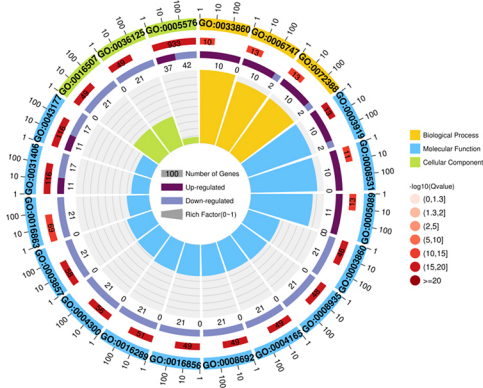**D**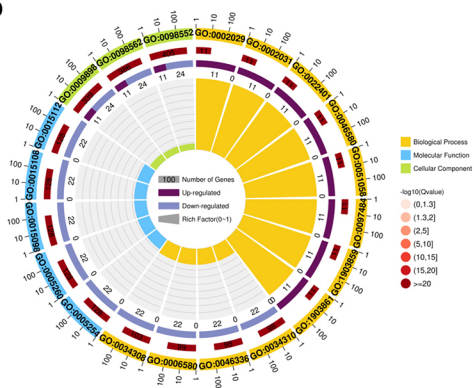

Supplement: Supplementary file 1 [file ijms-23-10515-s001.zip › Figure S1.pdf]

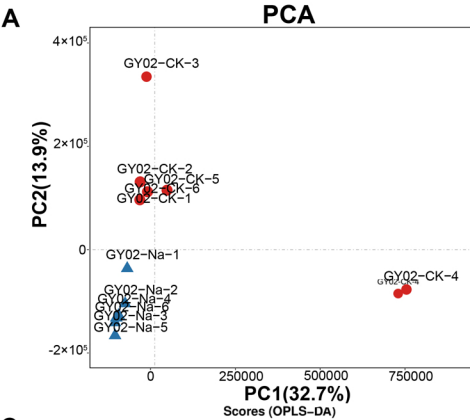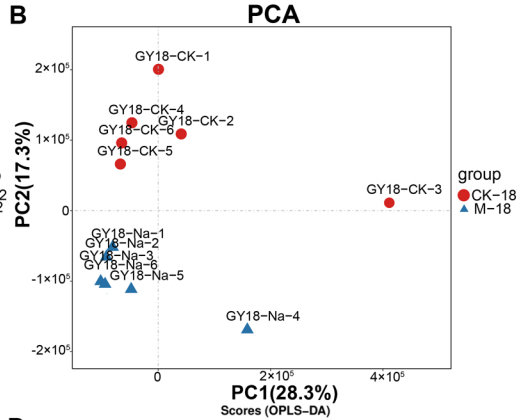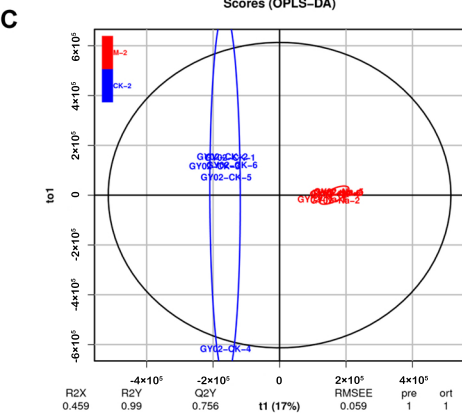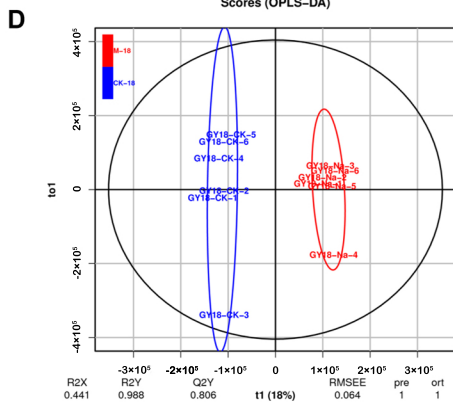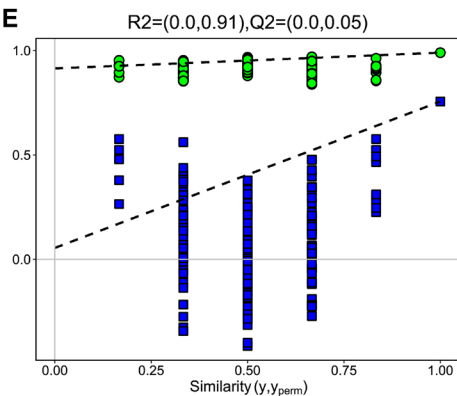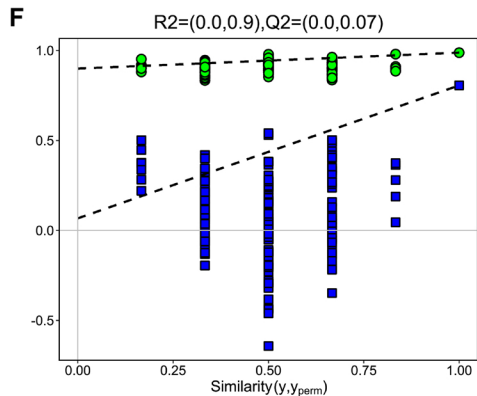

Supplement: Supplementary file 1 [file ijms-23-10515-s001.zip › Figure S2.pdf]
